# Familial Adenomatous Polyposis (FAP) Presenting as Iron Deficiency Anemia in a 33-Year-Old Female: A Case Report

**DOI:** 10.7759/cureus.24603

**Published:** 2022-04-29

**Authors:** Afrah Ali, Areesha Ahmad, Shah Taj, Shahid A Qaudeer, Syed E Ahmed

**Affiliations:** 1 Medicine, Lake Erie College of Osteopathic Medicine, Bradenton, USA; 2 Oncology, Lake Erie College of Osteopathic Medicine, Bradenton, USA; 3 Hematology/Oncology, Florida Cancer Specialists & Research Institute, Sebring, USA

**Keywords:** familial adenomatous polyposis, malignant colonic polyp, apc gene, iron deficiency anemia (ida), colon cancer and colon polyps

## Abstract

Iron deficiency anemia is a common clinical concern in women of reproductive age. It presents as microcytic anemia and can be due to a limited number of causes including bleeding, malabsorption, intravascular hemolysis, or a mechanical heart valve. Familial adenomatous polyposis (FAP) is an inherited autosomal dominant disorder due to mutation in the adenomatous polyposis coli (*APC*) gene that can cause iron deficiency anemia due to GI malignancy, most notably colon cancer. Variation of mutations within the *APC* gene can cause different forms of FAP, such as Gardner syndrome. This syndrome presents with epidermoid cysts typically in unconventional locations such as the face, scalp, and extremities, as seen in our patient.

We report a presentation of FAP in a 33-year-old Caucasian female who initially presented with iron deficiency anemia, hematochezia, and weight loss. Colonoscopy revealed hundreds of polyps within the colon, with two that were biopsied and reported as tubulovillous adenoma. The patient underwent a robotically assisted laparoscopic total proctocolectomy with ileal pouch-anal anastomosis, as well as a diverting loop ileostomy, and was given pain medication. She was referred to genetic counseling for her daughters and herself, which revealed a pathogenic variance in the *APC* gene.

## Introduction

Iron deficiency is the most prevalent nutritional deficiency seen across the globe and accounts for more than half of the cases of anemia [1]. The diagnosis of iron deficiency anemia is made by complete blood count (CBC) findings of low iron stores with a hemoglobin level two standard deviations below normal for the appropriate age and sex [1]. In women of reproductive age, the most common etiology is heavy menstruation causing high blood loss. Whereas, in the geriatric population of both men and older women, blood loss from the gastrointestinal (GI) tract is the most significant contributor to iron deficiency [2]. Contrary to what is more commonly clinically seen, in the case of familial adenomatous polyposis (FAP), iron deficiency anemia is caused by GI malignancy in a young-aged patient population.

FAP is an autosomal dominant genetic disorder characterized by hundreds to thousands of adenomas diffusely throughout the GI tract, with the potential to become malignant. It is caused by a germline mutation in the adenomatous polyposis coli (*APC*) gene on chromosome 5q21 [3]. Most cases have shown an inevitable progression to colorectal carcinoma by the approximate age of 30-40 years [4]. While most cases do progress to malignancy, FAP is responsible for only <1% of all colorectal cancer cases [5]. Within FAP, there are some commonly known subdivisions of the disease including Gardner syndrome, Turcot syndrome, and attenuated FAP [4]. The severity and different subtypes of the disease have been shown to be correlated with the location of the mutation on the *APC* gene [6].

Clinical symptoms are unlikely to be seen until the adenomas are large and numerous enough to cause rectal bleeding and anemia, which is most often noted in late adolescence or early adulthood [7]. Some nonspecific symptoms that can be noted include bowel changes, abdominal pain, abdominal masses, and weight loss, but they are not always seen. Diagnosis is largely based on suggestive family history, symptomatic history and physical findings, and bowel endoscopy or colonoscopy [7].

It is possible for patients with FAP to remain asymptomatic except for incidental findings of iron deficiency anemia. This may initially be masked by other clinical conditions including but not limited to heavy menstruation, pregnancy, and internal hemorrhoids.

## Case presentation

A 33-year-old female initially presented to her obstetrics and gynecology physician post-cesarean section. She was found to have persistent anemia with symptoms including fatigue and pica. She also noted years of intermittent hematochezia attributed to her history of hemorrhoids. She was referred to undergo an esophagogastroduodenoscopy (EGD), and to oncology for further evaluation. Her EGD at the time demonstrated multiple pedunculated polyps spanning the cardia, fundus, and body, which were all removed.

Upon further evaluation during her initial encounter with her oncology physician, the patient revealed a recent weight loss of 50 pounds, which she attributed to stress. Her past medical history revealed depression, internal hemorrhoids, and polycystic ovary syndrome (PCOS), for which she took metformin. Her surgical history revealed cesarean sections and surgical removal of epidermal inclusion cysts on bilateral lower extremities. Her family history was significant for liver cancer in her father and lung cancer in her mother. A physical exam at that time showed no abnormalities. A CBC revealed microcytic anemia with reactive thrombocytosis but was otherwise unremarkable. The patient was referred to undergo a colonoscopy for further evaluation of possible underlying malignancy contributing to anemia and significant weight loss.

**Figure 1:**
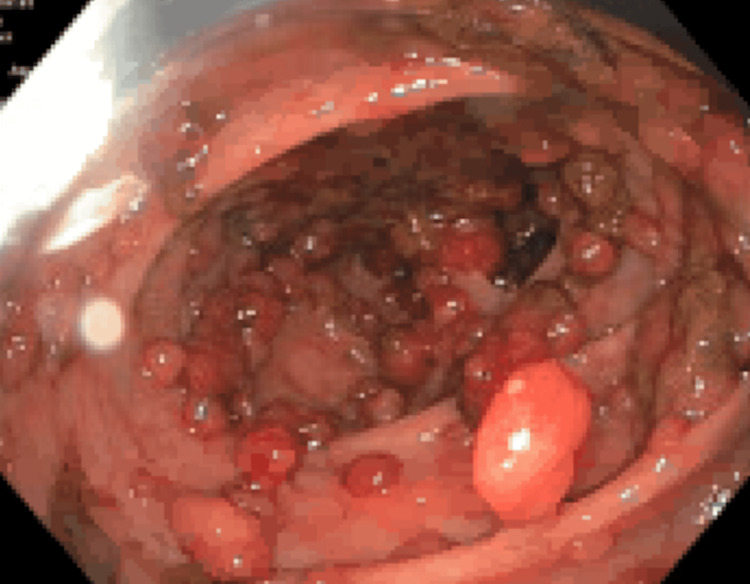
Transverse colon per colonoscopy

**Figure 2:**
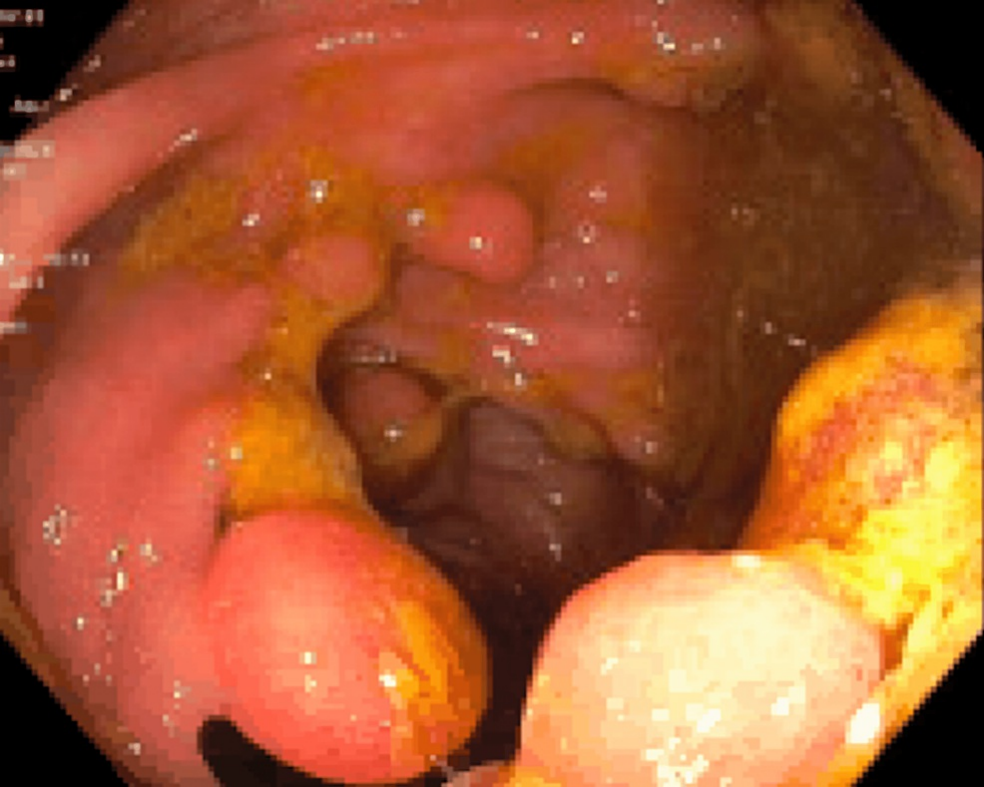
Sigmoid colon per colonoscopy

**Figure 3:**
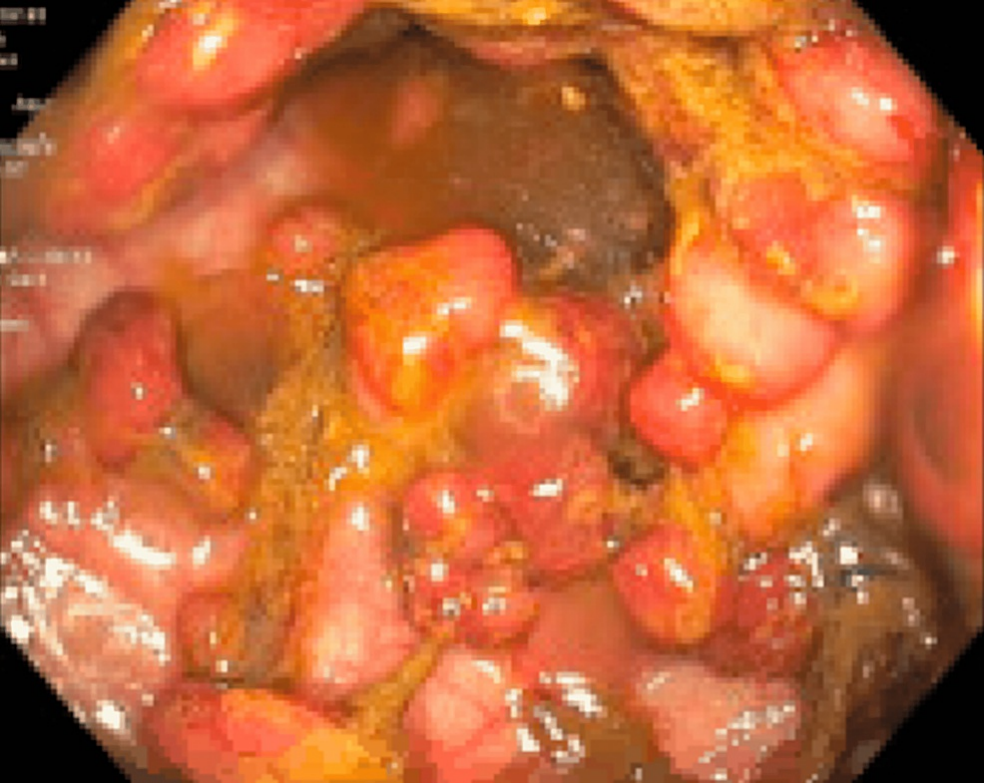
Rectum per colonoscopy

Colonoscopy revealed hundreds of polyps involving the entire colon, as shown in Figures 1-3. Two separate polyps in the sigmoid colon were biopsied, and pathology revealed a tubulovillous adenoma with high-grade dysplasia. She was deemed to have FAP and was referred for surgical resection.

**Figure 4:**
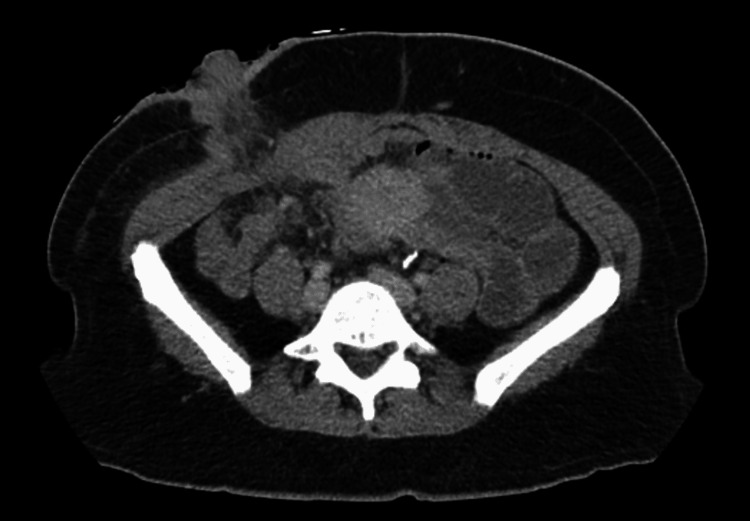
CT of the abdomen with contrast post-robotic total proctocolectomy with diverting loop ileostomy

She underwent a robotically assisted laparoscopic total proctocolectomy with ileal pouch-anal anastomosis, as well as a diverting loop ileostomy, as seen in Figure 4. Pathology at the time revealed well-differentiated invasive adenocarcinoma of the sigmoid colon, measuring 3.2 cm, infiltrating into but not beyond the muscularis propria. The surgical margins, terminal ileum, and appendix were found to be negative. A total of 87 lymph nodes were examined and found to be negative for malignancy. She was diagnosed with T2N0 colon cancer. Due to negative margins and lymph nodes, it was decided she did not need postoperative chemotherapy.

The patient was discharged postoperatively with pain medication and followed up in the oncology office on postoperative days seven and 19. On both follow-up evaluations, she was noted to be recovering well from surgery. Her incision site was observed to be well healing, clean, dry, and intact, with no issues with her ostomy. She was referred to genetic counseling for herself and for her two daughters to evaluate for FAP. The results of her diagnostic testing confirmed pathogenic variance in the *APC* gene. She was found to have a sequence change at exon 16, which creates a premature translational stop signal in the gene, which has been previously seen in individuals with FAP.

## Discussion

It is imperative that every physician who encounters a patient completes a thorough history and physical exam. The microcytic anemia seen in this premenopausal woman was attributed to a typical presentation post-cesarean section. However, after treatment, the patient’s obstetrician noticed that it was not resolving and referred the patient to oncology. It was not until the patient’s presentation at the oncology office that the patient’s hematochezia and weight loss were revealed. In this presentation of microcytic anemia, the cause was eventually isolated to GI bleeding secondary to polyps. However, the delay in diagnosing the cause of the patient’s anemia is noteworthy to discuss.

FAP is most often diagnosed based on clinical symptoms. Most patients present with intestinal symptoms such as lower GI bleed and diarrhea, whereas other non-specific symptoms such as abdominal pain, fatigue, and bloating are mainly reported by patients older than 40 years [8].

Gardner syndrome is a form of FAP. The mutation occurs on the *APC* gene on chromosome 5q22 and is inherited in an autosomal dominant fashion [9]. The manifestations of Gardner syndrome typically present around 25 years. GI polyps begin to form around puberty, and if not detected, can progress to colorectal carcinoma [8]. The noncutaneous features of Gardner syndrome include greater than a hundred colorectal polyps that progress to colon adenocarcinoma, osteomas, and pigmented lesions found in the eye [9]. Gardner syndrome is diagnosed with the following guidelines: 100 or more colorectal polyps or fewer than 100 polyps with a significant family history of Gardner syndrome, osteomas, and soft tissue tumors such as epidermal inclusion cysts and dermoid tumors [10].

This patient’s surgical history of epidermal inclusion cysts is the main manifestation of the cutaneous features of Gardner syndrome. The epidermoid cysts are typically seen in unconventional locations such as the face, scalp, and extremities [10]. This patient presented to a general surgeon with asymptomatic epidermoid cysts on bilateral lower extremities with no signs of urticaria, inflammation, or rupture, which can also be present [11]. This presentation could have been further investigated to potentially diagnose Gardner syndrome early on.

Additionally, patients with Gardner syndrome are more likely to develop other carcinomas of the thyroid, central nervous system, liver, and adrenal gland. Likewise, the recurrence of colorectal cancer is 30% in 20 years [12]. Therefore, regular follow-up and surveillance by oncology are vital in these patients. It is also imperative that direct family members, such as the children in this patient’s case, are tested for FAP. At the age of 35 years, 95% of patients will have polyps that rapidly increase [11]. Regardless, the overall prognosis of Gardner syndrome, if diagnosed early on, is excellent with 100% in five-year survival rates for patients who have a proctocolectomy [12].

## Conclusions

Ultimately, this report discusses a young female patient presenting post-cesarean section with iron deficiency anemia, a finding that can be initially dismissed as non-alarming due to this patient’s post-surgery status and history of bleeding hemorrhoids. The presence of epidermal inclusion cysts is usually addressed as a cosmetic issue with the only complication of note being infection. However, in conjunction with iron deficiency anemia, it can be indicative of unique pathology such as Gardner syndrome. Undiagnosed Gardner syndrome ultimately progresses to diffuse colorectal carcinoma.

To reiterate, it is imperative as medical professionals to constantly question and explore symptoms patients present with and keep in mind uncommon pathology that may be associated with relatively unconcerned findings initially.
